# Hospital and Institutional News

**Published:** 1911-10-21

**Authors:** 


### HOSPITAL AND INSTITUTIONAL NEWS.

### THE NEW WING AT THE GLASGOW INFIRMARY.

The handsome and commodious new Pavilion at the Glasgow Western Infirmary, which contains a fine clinical research laboratory, was opened with due formality by Sir James Barr on October 13. The new wing being practically a continuation to the south of the portion which was erected four years ago, its architectural features are in entire consonance. Messrs. John Burnet and Son are the architects for the whole of the buildings in collaboration with Dr. D. J. Mackintosh. The new wing has a frontage of 370 feet and is four storeys in height, with a white freestone front. Three wards 114 feet long, 28 feet wide, and about 14 feet high, accommodate twenty-four patients each. They are tiled to a height of 6 feet 6 inches and plastered above, the plaster being enamelled. The division between the observation and the main wards is of double glass, flush both sides to facilitate supervision. Two wards containing twelve and eight patients respectively are provided for the special treatment of skin diseases, and special baths have been provided. The new clinical and chemical laboratories, which form part of the scheme, will be under the directorship of Dr. Carl H. Browning, who was formerly lecturer on bacteriology at the Glasgow University and assistant pathologist to the infirmary. When the managers contemplated building the south-west wing they were greatly assisted by the generous offer of Mr. William Robertson to provide and equip, at a cost of £8,500, a large surgical ward of twenty-two beds, which had all the latest improvements, including a wide balcony to the south for the use of convalescent patients. But Mr. Robertson's liberality did not stop there. He promised a further sum of £3,500 to provide an operating theatre in a new building. One of the wards now in the new wing is to be called the M'Call Anderson Memorial Ward in grateful acknowledgment of Sir Thomas M'Call Anderson's many services, and in recognition of his distinguished career and the high regard in which he was everywhere held; and the other the A. B. Buchanan Memorial Ward, to associate it with the name of Sir Thomas M'Call Anderson's colleague in the work of the Hospital for Skin Diseases in the early years of its existence. Dr. A. B. Buchanan was the son of Dr. Andrew Buchanan, Professor of Physiology in Glasgow University from 1839 to 1876. The clinical laboratory is the gift of a generous friend, who resolutely refuses to allow his name to be disclosed.

### SIR JAMES BARR ON THE INSURANCE BILL.

The utterances of Sir James Barr are always distinguished by a certain breeziness and originality of outlook and of expression. Not so long ago he was forecasting a future in which the surgeon would be an extinct species, owing to the expansion of preventive medicine having abolished all needs for his services, though it is true the surgeons do not seem to have been greatly alarmed at these predictions. In his address on the occasion of the inauguration of the new wing, he once more took as his theme the preventive medicine of the future. Dealing with tuberculosis, he complained that at present no serious attempt had been made to raise up a race immune to the disease. Tuberculin dispensaries and sanatoria, he complained, will never stamp out the disease; but he took comfort that in Glasgow "the vision" (of the inhabitants) "is not obfuscated by the erroneous methods of the propagandists." He advocated improving the dwellings of the poor and their conditions of life, and greater precautions to ensure a pure milk supply. With these aims The Hospital, as its readers are well aware, has always been in sympathy; but surely Sir James does not advocate that nothing should be done for those who have already contracted tuberculosis, and everything for the protection of those who have not. Even from the view point of prevention alone, patients with active disease must be treated, because they are the foci whence new cases arise. It is of little use to elaborate a pure milk supply if no care is to be taken to cure or to segregate in sanatoria those who are distributing infective material broadcast in their sputa. In the concluding paragraphs of his speech, Sir James Barr dealt with the problems of malingering and swindling, which he regards as certain to arise out of the National Insurance Bill. The Compensation Acts have been, as the medical profession well knows, fertile sources of these undesirable qualities; and we think Sir J. Barr is probably right in asserting that the mischief will be greater still under the new Bill than even under the Compensation Acts. This is an aspect of the Bill which should certainly engage the attention of the medical deputations which are discussing the whole matter with the Chancellor of the Exchequer. The local Press reports Sir James as stating that "instead of preventing disease they were going to encourage it by the new Insurance Bill, which would lead to any amount of malingering and swindling. In that respect it would be much more pernicious than the Workmen's Compensation Act. Some thought that it would ruin the finances of the hospitals, and consequently hamper their work, but whether it did so or not it would not lessen the necessity for hospitals. It would impair independence, increase sickness, and hasten the degeneracy of a spoon-fed race. It was a great step in the downward path to Socialism and degeneracy. It was directly contrary to the manly, fearless, independent spirit which had hitherto characterised the Scottish nation. It would lower the standard of medical education and prevent intellectual young men from entering the medical profession. Possibly these evil forebodings might not be realised, and he hoped they would not." With the last sentiment every one of us will cordially agree.

### CLAIM AGAINST A HOSPITAL.

A curious case has just been decided in the Court of Appeal in favour of the Kidderminster Infirmary, whose Governors were sued by a former house surgeon there, Dr. Curtis, for injuries sustained while using the *x*-ray apparatus installed in the infirmary. The case is of great interest to all hospital boards, and should be carefully studied in connection with the article which appears on p. 75 of this issue concerning the liabilities of hospitals and the ways of insuring against them. It seems that the house surgeon and the visiting surgeon decided to undertake the treatment of ringworm with the Röntgen rays; but neither knew the correct exposure. The house surgeon volunteered to experiment upon his own arm, with the result that too long an exposure was given and a burn resulted. The action failed, both in the County Court and in the Court of Appeal, because it was clear that the doctor voluntarily offered to submit himself to the rays, and because the correct means of timing and judging exposure could quite easily have been ascertained if the trouble had been taken to find it out. There is one more point in the case as summarised by the Master of the Rolls which is worthy of attention. It is that the Court assumed that the house surgeon is a workman within the meaning of the Workmen's Compensation Act, though this question did not arise on the appeal. That this assumption is sound can hardly be doubted; and considering the frequency with which ill-health attacks hospital residents, owing to the conditions of their employment, it is astonishing that so little is heard of compensation demands and litigations. All the same, the risk is one which all hospitals will be well advised to insure against in the future.

### BIRMINGHAM HOSPITAL SATURDAY FUND.

The satisfactory result of the collections for the Birmingham Hospital Saturday Fund is distinctly encouraging, especially at the present time, when a very considerable extension of its work is now being undertaken in the erection of a new home for consumptives. Even without several contributions not yet paid in the total of the fund for 1911 amounts already to £21,463, which is an excess of £1,212 over the total for 1910. Sir Hallewell Rogers, Chairman of the Fund, pointed out that this sum was none too much when the expenses in connection with the new sanatorium were considered. This new home for consumptives is being erected on a site at Romsley Hill, about ten miles from the city, and will form a memorial to Sir William Cook. The foundation-stone was laid on October 14 by the Lord Mayor of Birmingham.

### THE NEW MONTGOMERY COUNTY INFIRMARY.

The new county infirmary which has been built at Newtown was opened on October 11 by Mr. David Davies, M.P., who has contributed half the cost of the building. The site has been presented by Mr. and Mrs. Powell in memory of their daughter, and a large proportion of the remaining cost was collected in small amounts from the poorer classes. The building cost £6,100. At the same gathering Sir Watkin Williams Wynn, Lord Lieutenant of the county, laid the foundation-stone of a new Tuberculosis Dispensary—the first in North Wales. The total cost of the erection of this much-needed dispensary will be borne by Mr. David Davies. When complete it is intended to place it at the disposal of the national campaign against tuberculosis.

### THE GRIMSBY HOSPITAL COMMITTEE.

A large and influential meeting of the Committee of Management of the Grimsby Hospital was held on October 11 to elect a new chairman. Mr. J. F. Wintringham has felt himself obliged to retire from this office, which he has so successfully filled for over six years, and the task of finding a successor was resolved by the election of Councillor R. W. Roberts, J.P., the Deputy-Mayor. It is no light thing for professional and business men to give up their time so freely as the exigencies of a hospital chairmanship demand, and we can well appreciate that the eulogistic speeches made by the Earl of Yarborough and by Lord Heneage in reference to the past work of Mr. Wintringham were but the expression of the opinion of all those who had worked with the retiring chairman. Mr. Wintringham's services, both as honorary secretary and as chairman, have been of lasting benefit to the hospital, and it is a pleasure to know that these offices were also held by his father, so that in electing Mr. Wintringham to be a vice-president of the institution the subscribers are showing their appreciation of the valuable services rendered to their hospital by two such assiduous chairmen in the same family.

### HOUSE-FLY DANGERS AND THE REMEDY.

The Gresham Professor of Physic, Dr. F. M. Sandwith, in a course of lectures at the City of London Schools, Victoria Embankment, during four evenings of October, spoke on "Flies as Carriers of Disease." The lectures were very well attended; several medical men and a good number of nurses in uniform were present. Dealing with the old theory of spontaneous generation, the Professor said Pasteur definitely proved that life could only proceed from life, and within the last few years we had learned that insects and other low forms of animal life play an important part in spreading disease. The house fly, although generally considered simply as a nuisance, should really be treated as a pest, for it is a real danger. After an interesting description of the structure and habits of the house-fly it was demonstrated how bacilli may be carried within and outside the fly, thus spreading summer diarrhœa, typhus, cholera, tubercle, etc. Sanitary authorities should circulate information and call attention to the danger of flies. The following is recommended as efficient for destroying flies: ℞ formaldehyde ʒij., sac. alb. ℥j., aq. ad Oj. In view of recent criticisms Dr. Sandwith states that to be effective it must be a 2 per cent. solution, *i.e.*, 1 in 50. This is the precise strength, for the flies will avoid it if it is too strong. A little sugar should be added to the solution with milk to attract them. This solution has been used for years in lavatories with success, despite all assertions to the contrary. Some flies perish in the saucer, but many die afterwards and will be found on the floor. The solution should be placed in saucers or other suitable vessels where flies are prevalent. Good commercial samples of formaldehyde (not stale) should be used, for old stock is of no use and means failure. The solution must be changed every day, as the formaldehyde gas readily volatilises in the open vessels. It is of the first importance to locate the breeding grounds and to destroy them.

### THE SAMARITAN HOSPITAL FOR WOMEN.

After considerable delays arrangements have been completed for the installation of a house surgeon at this Marylebone hospital, and the selected candidate commenced his duties on October 16. It seems extraordinary that a hospital where so much and such good work is performed should have contrived to do without any resident qualified officer for so many years; but the explanation no doubt is to be found in its near neighbourhood to Harley Street and the vicinity, so that the visiting staff can always easily be summoned in any case of emergency. Of the wisdom of this present step there can be little doubt; though apparently there was considerable division of opinion about it amongst the medical staff, and it required the authority and influence of a hint from the King's Fund to turn the balance in favour of this most salutary and necessary reform. The new house surgeon is Mr. Huyssen, who comes from Guy's Hospital, and he will have at first to be rather tactful in dealing with the responsibilities of a new office the creation of which did not meet with unanimous acceptance.

### THE GREENWICH NAVAL MEDICAL SCHOOL.

The fact that the Government has now issued the full regulations for the revised Naval Medical Service in terms of the recommendations made recently by the departmental committee is commented upon in a leading article in this issue. Institutional workers will be interested in the new scheme because it brings into prominence one of our most interesting metropolitan institutions, the Seaman's Hospital at Greenwich. Under the new regulations this institution is to become the real training school of the naval surgeon. It already is a training school for a large majority of naval medical men, but in a few years' time it is likely that it will absorb the greater number of candidates for the naval examinations who now, notwithstanding the admittedly high claims of the Dreadnought School, patronise other teaching centres. We heartily congratulate everyone concerned upon the step that the Admiralty has taken in selecting the Dreadnought as the new school. The high standard of its teaching—second to no other school in London, as witness the fact that men from every other teaching hospital flock to Greenwich to take coaching courses for the higher examinations—is so well recognised that it need not be dilated upon here. Students under the new regulations will have the added services of two new professors who will hold the rank of Fleet Surgeons and will lecture on bacteriology and pathology and on naval hygiene respectively. Arrangements have been made to enable such students to board and lodge at the Royal Naval College, where there is a large and well-maintained mess for officers of the various branches of the Royal Navy and Royal Marines. This arrangement will serve admirably for the present, but there can be little doubt that in course of time, when the school grows, it will become necessary to build a residential college for the benefit of the graduate students. Such a culmination will be in the interests both of the hospital and of the service, and will fittingly serve to show to the public the importance of the work that is being carried on at Greenwich.

### THE HOSPITAL SHIP OF THE ORDER OF MALTA.

The Naples correspondent of the *Stampa* states that the Order of Malta, which renders service to the army in war time, has been requested by the Government to prepare and equip the warship *Regina Margherita* required by the State under present circumstances. The preparation has been terminated and this floating hospital has now started for the theatre of war. There are on board some 300 beds adapted for hospital use, together with all the ordinary necessaries for sanitary service. The direction has been confided to the Chevaliers of the Order, Prince Di Sonnino, Don Prospero Colonna, and Gritti Morlacchi. The inspector for this particular mission is the Chevalier of Justice Cugia di Sant' Orsola; the head of the medical staff is Doctor Achille De Fabi, the medical assistants are Doctors Mazzatelli, Giani, Franceschini, and Roncoroni. A responsible official and forty sub-officials and male nurses have been told off for the sanitary service. To complete the pious work, six Sisters of the Institute of Charity of San Vincenso de Paoli have embarked also. This is the first expedition that the Order of Malta is making, but some others will follow. His Highness the Prince Grand Master has arranged that the vast *sala* which the Order maintains in the Hospital for Incurables shall be kept in readiness for all the wounded who can come to Naples, as was done in the time of the war in Africa.

### THE PROGRESS OF THE NATIONAL INSURANCE BILL.

When the House of Commons meets next week it should be possible to obtain a fair idea of the prospects of the National Insurance Bill. During the week opposition seems to have grown, but, on the other hand, the Chancellor's emphatic statements on Saturday last leave little room for doubt that the Bill will be passed before the end of the year, if in any way possible. The Conference between representatives of the British Medical Association and of the Friendly Societies was resumed on Monday, and after a six hours' sitting was again adjourned. If Mr. Lloyd George comes to terms with the different interests concerned the discussions in the House of Commons will naturally be shortened, but if no agreement is arrived at, the Government may place a time limit on the discussions.

### THE SESSION AT CENTRAL THROAT HOSPITAL.

The inaugural lecture of the winter session at the Central London Throat and Ear Hospital, Gray's Inn Road, will be delivered by Dr. Watson Williams on Friday, October 27, at three o'clock. The title of the lecture will be "The Influence of the Upper Air Tract on Respiration." On the same evening will take place at the Trocadero the annual dinner of the medical staff, Dr. Andrew Wylie presiding.

### THE FINSBURY COUNCIL OF SOCIAL WELFARE

A Conference will be held on Thursday, October 26, at 4.30 p.m., in the Finsbury Town Hall, Rosebery Avenue, in connection with the work of the Finsbury Council of Social Welfare. The Council is making an earnest appeal for funds to carry on the work of arresting the progress of consumption by retaining the services of the health visitor, who has proved of the greatest assistance in advising and controlling phthisical patients in the borough. The conference has been called by the Mayor of Finsbury, who has secured the attendance of Sir Arthur Downes, M.D., the Senior Medical Inspector (Poor Law) at the Local Government Board; it is proposed to hold an informal discussion in which Sir Arthur Downes will take part.

### THE SCANDAL OF OUT-PATIENTS.

Our article on the subject of the overcrowding of the out-patient departments of the Metropolitan hospitals has attracted a good deal of attention and led to some discussion, which we welcome, since it shows that institutional workers are keenly alive to the dangers of the present system and anxious to obviate them if possible. We have been asked to inspect and report on several newly-constructed out-patient departments, which are claimed to be admirably managed and to possess the requisite amount of air space and through ventilation necessary in such buildings. While we are at all times willing to describe and discuss new arrangements and the merits of improvements effected at various institutions, we would point out to our correspondents that our indictment concerned those hospitals which have neglected to put their out-patient departments in order. It is much more necessary, therefore, to describe and comment upon these mismanaged departments, and we hope to be able to do so very shortly when we have further material, in the shape of air analyses, photographs, and details of cases, before us. For the present we content ourselves by drawing attention to the danger of infectious cases in the out-patient department. Under the system which prevails at many hospitals new patients are allowed to mix with old, and no attempt is made to differentiate between ordinary acute, or chronic conditions and cases which may possibly be a focus of infection to those who come into contact with them. Our interest in this aspect of out-patient mismanagement has been roused by the recent epidemic of scarlet fever in North London, in view of the fact that it seems highly probable that some of the children were infected in the out-patient department of certain institutions. We are quite aware that the system of segregation which we advocate is difficult to carry out in practice, since it entails the supervision of an expert nurse or attendant, but we do not think that the difficulties in the way are insurmountable. Certainly the good results of the system ought to outweigh the theoretical objections to it which have been so often advanced. In the interests of the staff alone it is highly desirable that the division between old and new patients should be rigidly enforced. A separate entrance should be provided for new patients, at least on the medical side, with proper isolation rooms attached for the use of suspected cases of infectious disease. Provision for such a system has been made, we are glad to notice, in the plans for the out-patient departments of several of our newest hospitals—notably the new King's College Hospital. In a future article we shall deal with this question of infectious cases in the out-patient department at length.

### THE LATEST HOSPITAL SCANDAL.

At a recent inquest a coroner commented strongly on the fact that a very badly-injured patient had been sent away from Guy's Hospital to the London Hospital because the former institution declared that it had no bed available. The public Press, notably such organs as delight to make the flesh creep with the same eager vindictiveness that characterised the Fat Boy, has commented in even stronger terms on this want of organisation whereby an admittedly grave case is sent away, at a certain risk to the patient, owing to the fact that no spare beds are available. To hospital workers we need not outline the reasons which, unhappily, prevent a large hospital such as Guy's from being in a position, at any time, to admit every serious case that comes to it. There are undoubtedly reasons which justify the refusal of an accident case under certain conditions, but it is possible that in certain institutions too much weight is attached to them. Even at the risk of adding to the number of "dead beds," and therefore risking the appearance, in the annual report, of a lower figure for the "number of beds occupied daily" than has hitherto been the case, it is desirable that a few spare beds should be kept open and reserved exclusively for accidents. At Guy's there are two large male accident wards and a certain number of female accident beds available in the surgical wards, but the number of female accident beds is too few in comparison with the total number of beds in the institution. We offer no comment on the special case which has led to the present discussion in the lay Press, since we have not heard the explanation given by the authorities of the hospital. But the public may undoubtedly contrast the want of accommodation for accident cases at Guy's with the ampler provision made for such cases at the London.

### FEVER STATISTICS IN LONDON.

The annual report of the Metropolitan Asylums Board contains, as usual, a medical supplement full of statistics dealing with the cases under treatment during the year. There is much of interest in this section of the report concerning the various fevers with which the medical staff has to deal. Thus it appears that seven laparotomies were performed for perforation in typhoid fever, without a single recovery, though one of the patients survived thirty-four days and another one twenty-two. The summary of the last ten years' experience shows that the recovery-rate after operation has been 7.5 per cent., which shows how terribly fatal this complication still is. In the hospitals of the Board every facility for prompt diagnosis and operative treatment exists, so these figures are doubly significant of the prognosis of perforation. The other most notable contribution is by Dr. J. D. Rolleston on the blood-pressure in diphtheria. This was found to be subnormal in 35.1 per cent., the extent and duration of the depression having as a rule a direct relation to the severity of the faucial attack. In the majority of patients the highest readings were obtained in the first, and the lowest in the second, week of the disease. In laryngeal cases disproportionately high readings were obtained, especially when dyspnœa was bad enough to require operation. Tracheotomy was then followed by immediate and steep fall of blood-pressure. In early paralysis the pressure falls; but in late paralysis, even when extensive, it is usually not altered. There is little tendency to be affected by the early serum phenomena. Finally, the author concludes that in diphtheria, as in other acute diseases, sphygmomanometry has little practical significance, though it is of considerable theoretical interest.

### A NOVEL INVITATION CARD.

The Western Infirmary, Glasgow, has made a new departure by giving on its invitation card illustrations of the new ward pavilion with interior views of the new memorial wards and the clinical laboratory. This card has caused a surprising amount of interest throughout Glasgow and the neighbourhood. It produced a record attendance at the opening of these new buildings on the 13th inst. The idea is a good one and we hope it will be largely followed in future by other institutions. Then a collection of these cards of invitation might constitute a most useful file of information for reference and the encouragement of those who wish to make the institutions under their management up-to-date and widely appreciated.

### LONDON SCHOOL OF TROPICAL MEDICINE.

The Annual Dinner of the London School of Tropical Medicine was held at Prince's Restaurant, Piccadilly, on Tuesday night, under the presidency of Dr. M. Cameron Blair, Senior Sanitary Officer of Northern Nigeria, a distinguished student of the school. Among those present were Sir Patrick Manson, Sir James Porter, Director-General of the Medical Department of the Navy, Surgeon-General A. M. Branfoot, President of the Medical Board of India Office, Captain A. W. Clarke, a member of the Board of Management, Dr. W. F. Daw, P.M.O. of British Guiana, Mr. James Cantlie, Dr. C. W. Daniels, Dr. H. B. Newham, Director of the School, Colonel A. Alcock, entomologist, Dr. R. T. Leiper, helminthologist, Dr. C. M. Wenyon, protozoologist, and about 100 past and present students of the school. The toast of "Prosperity to the School" was moved by Surgeon-General Branfoot, and responded to by the Chairman and Sir Patrick Manson; the toast of "The Guests" was moved by Captain Clarke and responded to by Sir James Porter.

### A FAMOUS DAVOS PRACTITIONER.

The best-known personality in the very self-contained community of Davos Platz has just passed away by the death of Dr. William Richard Huggard, M.D., his Britannic Majesty's consul at Davos. Dr. Huggard, an M.D. of the Royal University of Ireland, which in 1909 bestowed on him the honorary LL.B., having qualified in 1875, at one time was physician to the St. Pancras and Northern Dispensary, but his success was gained and his reputation established in Switzerland. He had resided there for twenty-five years, and in order to practise took the Swiss Federal Diploma in 1885. Two subjects interested him; first of all, insanity, which led to his writing various articles in medical and scientific journals in the early eighties, prominent among which were his "Definitions of Insanity" and "The Lunacy Law"; and, secondly, climatology in relation to phthisis. It was, of course, phthisical cases to which he devoted the greater part of his life, and he had, chiefly as consultant, a large following of patients in Davos. He was striking in appearance, being much below middle height, with a penetrating gaze and somewhat abrupt manner that always attracted attention in any assembly; but on the rare occasions when he was induced to speak in public his matter was in striking contrast to his delivery, which was halting and nervous. Living a somewhat secluded life in his flat, he had not a large circle of intimate friends, but they were very loyal and enthusiastic on his behalf, and his office of consul enabled him to help many an Englishman who, by some lack of knowledge of Swiss law, which regards as offences certain actions not cognisable by our English courts, had fallen into difficulties. It is worth while emphasising the influence he possessed both in English and Swiss circles, as the official obituary published of him in the *Times* was meagre in the extreme, and showed indeed no knowledge beyond what is contained in the Medical Directory. The world of consumptives is a world to itself, and many of those patients who in the course of the wanderings after dry air and sunshine spent a month or more in Davos Platz carried news of him to health-resorts in all parts of the world. Among the medical men whose names stand out especially in the annals of modern phthisical treatment Dr. Huggard has as individual a place as Dr. Walther or Dr. Carl Spengler, the latter of whom still commands in Davos an equally enthusiastic following. Dr. Huggard's knowledge and personal traits, apart from his consular office, gave him a position as the leader of the English community as interesting and unique in its way as that attained by John Addington Symonds, another "old Davoser," in the world of letters. It should be added that Dr. Huggard paid a prominent part in establishing the new Queen Alexandra Sanatorium for less well-to-do patients, the annual report of which was recently reviewed in these columns.

### THE ZENANA MEDICAL MISSION.

On Thursday next, October 26, the Lord Mayor will preside at the annual meeting of the Zenana Bible and Medical Mission at the Mansion House. The Mission may claim to have a decided institutional importance, since it possesses many hospitals and dispensaries, babies' homes, and leper asylums, which give ample opportunity for personal service on the part of no fewer than 395 workers. For sixty years it has carried on useful medical and educational work in India. Last year it dealt with more than 50,000 patients, and this year it is probable its scope will be much increased owing to the disasters in the way of famine and disease with which India has had to cope recently. The Mission needs help, both financial and in the way of service, and institutional workers who sympathise with its aim and policy cannot do better than support the Lord Mayor next Thursday.

### THE HANDLING OF BUTCHERS' MEAT.

A vigorous campaign is being pursued by the Pure Food and Health Society of Great Britain with the object of securing more healthful conditions in the sale of butchers' meat. It is unfortunately true that the public is not sufficiently impressed by the conditions under which meat is handled in the market, and the Society will be doing a useful work if it succeeds in obviating the almost nauseating state of affairs now existing. Anyone who has observed the carting and handling of carcases, and cares to study the conditions under which the meat is exposed for sale in shops, will agree with Mr. A. E. Moore that there is much justification for the Society's decision to bring the matter before the Government through their members on the Parliamentary Committee on Food Reform. This will be done if possible early in November, with the view to securing the introduction of a short Bill on the subject. The Society invites those who feel strongly on the matter to send a postcard to 20 Hanover Square, and thus strengthen the hands of the Society when they bring the data they have acquired before the Parliamentary Committee.

### NOTIFICATION OF PHTHISIS IN BRISTOL.

Last week the Bristol City Council ratified the recommendation of the Health Committee to the effect that tuberculosis should be included among the notifiable diseases. The opposition against the notification of phthisis has during the last few years sensibly diminished, and it has been recognised that, although notification is one only of the weapons available in the fight against tuberculosis, it is a very important one. To know the distribution of the enemy must always be an advantage, and one no longer hears anything of the objection formerly raised regarding "social ostracism." As a means of educating the public and carrying out the principle of the greatest good to the greatest number, notification has advantages which outweigh its alleged disadvantages.

### THE NATIONAL INSURANCE BILL AT LLANDAFF DIOCESAN CONFERENCE.

At the Llandaff Diocesan Conference, at Newport (Mon.), last week, the greater part of an afternoon was devoted to a discussion on the National Insurance Bill, following a motion by Mr. J. A. Lovat Fraser, to the effect "that the Diocesan Conference desires to express its sincere sympathy with the general objects of the National Insurance Bill now before Parliament." Mr. Lovat Fraser, who was supported by a number of the clergy, had no difficulty in eliciting sympathy for the general objects of the Bill, but other speakers, notably Mr. Vicary, of Aberdare, and Mr. Brett, of Cardiff, spoke very strongly, as representing Friendly Societies' interests, whilst the Rev. W. D. I. Mackintosh made a strong appeal that due consideration should be afforded to the claims of the Voluntary Hospitals of the Kingdom, as in his opinion it would prove calamitous if the resources of these great "Christian Institutions" should be weakened, as they undoubtedly would be under the proposed conditions of the Bill. Eventually Mr. Fraser's motion was adopted, with the following rider, which was seconded by Mr. Mackintosh: "That in view of the vast importance of the Bill, the Conference strongly urges that further time should be given for its consideration."

### NEW COTTAGE HOSPITAL FOR SKEGNESS.

On a site given by the Earl of Scarbrough, the first step was taken the other day towards the erection of a cottage hospital for Skegness and district. The foundation-stone was laid by Princess Marie Louise of Schleswig-Holstein amid the enthusiastic rejoicing of a large assembly. The approximate cost of the building will be £900, to which must be added about £300 for equipment. The hospital will contain two private wards, in addition to male and female wards and accommodation for nurses. The designs of Mr. F. J. Parkinson, of Blackburn, have been accepted. A cottage hospital has long been required in this growing and enterprising seaside resort, which, in spite of the greatness which has been thrust upon it by railway posters, needs must cater for the sick like the rest. The nearest hospitals being at Spilsby and at Boston, the local practitioners will doubtless welcome this very desirable acquisition so brilliantly inaugurated.

### THIS WEEK'S DRUG MARKET.

The improvement in business which set in a few weeks ago is maintained. Generally speaking, prices continue to tend upward, but there are no striking advances in value to record. Citric acid and citrates (including iron and ammonium citrate and potassium citrate) are dearer. Balsam tolu is still tending upward in price, as also are carbolic acid and cream of tartar. Ipecacuanha, oil of aniseed, and manna are dearer. Cloves are rather lower in price.

